# Headspace solid-phase microextraction comprehensive 2D gas chromatography-time of flight mass spectrometry (HS-SPME-GC × GC-TOFMS) for origin traceability of the genus *Hymenaea* resinites†

**DOI:** 10.1039/d3ra00794d

**Published:** 2023-05-09

**Authors:** Xiaopeng Su, Jing Yu, Zhaotong Shi, Yamei Wang, Yan Li

**Affiliations:** a Gemmological Institute, China University of Geosciences Wuhan 430074 China yanli@cug.edu.cn wangym@cug.edu.cn; b Hubei Engineering Research Centre of Jewellery Wuhan 430074 China

## Abstract

Differentiating the chemical compositions of resinite (amber, copal, and resin) is very crucial for determining the botanical origin and chemical compositions of the fossilised amber and copal. This differentiation also assists in understanding the ecological functions of resinite. Headspace solid-phase microextraction-comprehensive two-dimensional (2D) gas chromatography-time-of-flight mass-spectroscopy (HS-SPME-GC × GC-TOFMS) was firstly proposed and utilised in this research to investigate the chemical components (volatile and semi-volatile compositions) and structures of Dominican amber, Mexican amber, and Colombian copal for origin traceability, which were all produced by trees belonging to the genus *Hymenaea*. Principal component analysis (PCA) was used to analyse the relative abundances of each compound. Several informative variables were selected, such as caryophyllene oxide, which was only found in Dominican amber, and copaene, which was only found in Colombian copal. 1*H*-Indene, 2,3-dihydro-1,1,5,6-tetramethyl- and 1,1,4,5,6-pentamethyl-2,3-dihydro-1*H*-indene were abundantly present in Mexican amber, which were the critical fingerprints for the origin traceability of amber and copal produced by trees from the genus *Hymenaea* of various geological places. Meanwhile, some characteristic compounds were closely related to the invasion of fungi and insects; their links with ancient fungi and insect categories were also decoded in this study and these special compounds could be used to further study the plant–insect interactions.

## Abstract 2

Headspace SPME-GC × GC-TOFMS was used to analyze the volatile fraction of resinite. Marker molecules and PCA of VOCs’ aboundances allowed for distinction. To distinguish the different geographical origin of the genus *Hymenaea* resinite.

## Graphical abstract

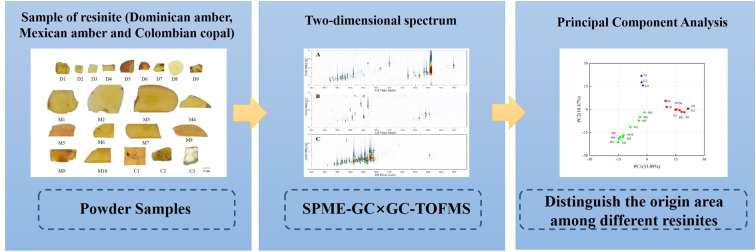

## 1 Introduction

Resinite (a fossil resin, including amber, copal, resin) is a complex biogenic polymer that originated from ancient plant resin buried underground through long-term various geological periods and fossilized over tens of millions of years of geological process.^1^ As a typical complex organic polymer with large molecules, it had undergone natural polymerization and fossilization.^2^ Resinite began to form with the increase in the aggregation degree of major organic components and the continuous escape of volatiles. The compositions of resinite were regulated by the ancient plant types from the source and the burial circumstances during the evolution process.^3,4^ Researchers could use the stable carbon and hydrogen isotopes of resinite to reconstruct the partial pressure of oxygen in the paleoatmosphere.^5,6^ Moreover, resinite also determines its paleoplants and their interactions with the surrounding environment.^7,8^ Therefore, it is necessary to thoroughly understand the chemical compositions of various kinds of resinite and the ancient plants while restoring the habitat of the ancient plants.

Resinite was classified into five types based on the structural characteristics of the original resin.^9^ The *Hymenaea* amber and copal belong to the *Ic* class, which was based on the polymerised lambdoid diterpenes and polymers of enantio-labdanoids that lack succinic acid.^10^ Dominican amber, Mexican amber, and Colombian copal were all produced by the extinct tree species of the genus *Hymenaea*,^11–15^ whose producing areas were geographically close to one another. Dominican and Mexican ambers were found in Miocene, and the Colombian copal was produced in the Pleistocene. However, the genus *Hymenaea* contains different tree species. Diterpenes are the dominant components in the non-volatile portion of *Hymenaea* amber, and the volatile fraction comprises sesquiterpenes that most often occur as hydrocarbons with some oxygenated constituents.^16^ Specific terpenoid skeletal types often characterise the taxa, such as particular families and genera.^17^ The volatile components of amber with low molecular weight easily escape from the amber and record detailed information of chemical components. Combined with multivariate statistical analysis methods, this approach can distinguish the places of origin of resinite.

The most common analytical technique used to differentiate the origin of resinite was gas chromatography-mass spectrometry (GC-MS). However, the previous methods for resinite pre-treatment (such as organic solvent dissolution, Soxhlet extraction, rotary evaporation, *etc.*) are tedious operation, long processing time, large consumption of organic reagents, and environmental pollution,^18^ and traditional spectroscopy examinations are difficult to detect the specific components of resinite, and the common one-dimensional (1D) GC-MS isolating and detecting methods also have defects such as limited isolation and detection efficiency, frequently missing date on volatile components'. What's more, the peak capacity and resolution of 1D GC-MS were low, the peak overlap was serious, and a series of co-outflow peaks often appeared on the chromatogram, which hinder the comprehensively and accurately identify its chemical composition.^19^ Wang *et al.* improved the above pretreatment technology and analysis method, carried out fine chemical composition analysis of the soluble components in Dominican amber with blue fluorescence, and firstly reported that 15-nor-cleroda-3,12-diene was a biomarker of Dominican amber, revealing that the ancient plant source of Dominican amber was *Hymenaea*.^20^ Although great progress had been made in the analysis of amber chemical components, other complex biomarkers have not been effectively resolved.

Comprehensive two-dimensional gas chromatography (GC × GC) with high peak capacity, high resolution, and high sensitivity, and has been widely applied in petrochemical engineering, environmental protection, and metabolites.^21–23^ Its “structural spectrum” is conducive to solving the difficulties and pain points in the superimposition of 1D chromatographic peaks, breaking through the technical bottleneck in the separation and analysis of complex organic components, and screening more abundant resinite biomarkers.^24^ In addition, comprehensive GC × GC is a novel method that has also been used to analyse the compositions of agarwood from different places of origin and identify the characteristic compositions of different agarwood samples.^25^ Several studies have also employed a TOFMS coupled to Py-GC × GC, and were able to identify organic molecules including biomarkers in three different classes of fossilized organic material.^26^ Besides, HS-SPME uses a coated fibre to extract the volatile components from the headspace of a sealed vial containing a sample and transfer them to a gas chromatography-mass spectrometer (GC-MS) for further identification and quantification.^27^ A previous study used the HS-SPME-GC-MS technology to analyse the volatile fraction of low molecular mass for identifying the Baltic and Romania amber.^28^ Furthermore, this technology was also used to analyse the organic constituents of American and African amber, copal and resin and explore their palaeobotanical origins.^14^ In order to improve the resolution of organic components in chromatography, our group has optimized the pre-treatment method of headspace solid phase microextraction (HS-SPME) in advance, and analysed Dominican amber, Mexican amber, and Columbia copal *via* GC × GC-TOF-MS, and rich information of organic compounds was obtained. Therefore, a novel method of HS-SPME-GC × GC-TOFMS suitable for resinite analysis is practical and feasible, which is expected to open the molecular structure window of resin maturity evolution, accurately screen the typical biomarkers in resinite, accurately and effectively determine the ancient plant source of resinite from the molecular structure level, and fill in the defect that previous qualitative understanding of plant source based on its internal inclusions (plant or insect inclusions) or the insufficient precision of plant source determined by 1D GC-MS method.

Hence, the sample pre-treatment technology of headspace solid-phase microextraction combining with comprehensive two-dimensional gas chromatography and time-of-flight mass-spectroscopy for characterisation and traceability of the genus *Hymenaea* resinite from various geographical origins were creatively proposed, which could detest 2–3 times as many compounds number as the traditional analysis method. The research aims to use HS-SPME-GC × GC-TOFMS to analyse the volatile components of various Dominican and Mexican ambers and Colombian copal produced by trees from the genus *Hymenaea* to screen the different volatile fractions of amber and copal for origin traceability.

## 2 Materials and methods

### 2.1 Resinite samples

Nine pieces of Dominican amber (D1–D9), ten pieces of Mexican amber (M1–M10) and three pieces of Colombian copal (C1–C3) were selected from the Guangzhou Gem Testing Centre, China University of Geosciences, Wuhan (Table 1). The origin has been tested according to spectroscopy and is reliable.^29^ All the selected specimens were internally homogenous and did not contain any organic inclusions.

**Table 1:** Sample descriptions of amber/copal used in this study

ID	Provenience	Sample type	Age (Ma)	Colour
D1	Dominican Republic	Amber	Miocene (20–15 Ma)	Light yellow, transparent, shiny
D2	Dominican Republic	Amber	Miocene (20–15 Ma)	Light yellow, transparent with dark inclusion, shiny
D3	Dominican Republic	Amber	Miocene (20–15 Ma)	Light yellow, transparent, shiny
D4	Dominican Republic	Amber	Miocene (20–15 Ma)	Light yellow, transparent, shiny
D5	Dominican Republic	Amber	Miocene (20–15 Ma)	Dark yellow, semi-transparent, shiny
D6	Dominican Republic	Amber	Miocene (20–15 Ma)	Dark orange, transparent, shiny
D7	Dominican Republic	Amber	Miocene (20–15 Ma)	Light yellow, transparent with dark inclusion, shiny
D8	Dominican Republic	Amber	Miocene (20–15 Ma)	Light yellow, transparent, shiny
D9	Dominican Republic	Amber	Miocene (20–15 Ma)	Yellow, transparent, shiny
M1	Chiapas, Mexican	Amber	Miocene (22.88 ± 0.90 Ma)	Light yellow, transparent, shiny
M2	Chiapas, Mexican	Amber	Miocene (22.88 ± 0.90 Ma)	Light yellow, transparent, shiny
M3	Chiapas, Mexican	Amber	Miocene (22.88 ± 0.90 Ma)	Dark yellow, transparent, shiny
M4	Chiapas, Mexican	Amber	Miocene (22.88 ± 0.90 Ma)	Light yellow, transparent, shiny
M5	Chiapas, Mexican	Amber	Miocene (22.88 ± 0.90 Ma)	Light orange, transparent, shiny
M6	Chiapas, Mexican	Amber	Miocene (22.88 ± 0.90 Ma)	Light yellow, transparent with dark inclusion, shiny
M7	Chiapas, Mexican	Amber	Miocene (22.88 ± 0.90 Ma)	Dark yellow, transparent, shiny
M8	Chiapas, Mexican	Amber	Miocene (22.88 ± 0.90 Ma)	Dark yellow, transparent, shiny
M9	Chiapas, Mexican	Amber	Miocene (22.88 ± 0.90 Ma)	Dark orange and yellow, transparent, shiny
M10	Chiapas, Mexican	Amber	Miocene (22.88 ± 0.90 Ma)	Dark yellow, transparent, shiny
C1	Colombia	Copal	Pleistocene (2.5–0.2 Ma)	Dark yellow, semi-transparent
C2	Colombia	Copal	Pleistocene (2.5–0.2 Ma)	Dark orange, dark inclusion, opaque
C3	Colombia	Copal	Pleistocene (2.5 ∼ 0.2 Ma)	Yellow, transparent, shiny

### 2.2 Conditions of headspace solid-phase microextraction

Three various samples (Dominican amber, Mexican amber, and Colombian copal) were crushed into powders using mortar and were mixed equally. Meanwhile, the samples of obtained powders were screened through a 50-mesh sieve. Next, 0.01 g of these powders were placed in a 20 mL headspace sample bottle. The powders' vials were placed on the heating device and equilibrated at 80 °C for 15 min isotherm. The SPME fibre, a 65 μm polydimethylsiloxane/divinylbenzene (PDMS/DVB) fibre initially conditioned at 250 °C for 15 min, was introduced and exposed to the headspace for 15 min.

After the sample preparation, the SPME device was immediately inserted into the GC × GC injector, and the fibre was thermally desorbed for 30 min at 250 °C. The fibre was reconditioned for 30 min in the GC × GC injector port at 250 °C to eliminate memory effects before changing the following sample.

### 2.3 Characterisations techniques

The GC × GC-TOFMS instrument is a Pegasus 4D system comprising a LECO time-of-flight mass spectrometer (St. Joseph, MI, USA) and an Agilent 7890A GC (CA, USA) equipped with a secondary oven and a quad-jet dual stage modulator. The 1D and 2D columns used for this study were Agilent HP-5ms column (5% phenyl 1% vinyl dimethyl polysiloxane, 30 m × 0.25 mm i.d. × 0.25 μm df) and DB-17HT column (50% phenylpolysilphenylene siloxane, 1.0 m × 0.1 mm i.d. × 0.1 μm df), respectively. The flow rate of the carrier gas (He, 99.999%) was kept at 1 mL min^−1^. The temperature of the first GC oven was initially maintained at 50 °C and subsequently heated at a rate of 1.5 °C min^−1^ to 200 °C. Then, the temperature was increased to 280 °C ramping at 10 °C min^−1^. The temperatures of the second GC oven and the remaining modulator were the same as in the first oven. The modulation period was 4 s with a hot pulse time of 1 s. The mass spectroscopy ion transmission line and ion source temperatures were 280 °C and 250 °C, respectively. Data were processed using ChormaTOF-GC version 4.51.6 (LECO). The temperature program was optimized based on our amber team (associate professor, Yamei Wang) previous work for the Dominican amber. 1 The volatile components of resinite samples contain a large number of sesquiterpenes components, including many isomers, which tend to overlap in the chromatogram. In the temperature program of this study, a better degree of separation was obtained by reducing the earlier heating rate.

### 2.4 Data processing

Previous studies^30,31^ provided the data analysis process of the amber extract compositions. The chromatograms for each sample were imported into the programme – The Automatic Mass Spectral Deconvolution and Identification System (AMDIS), which automatically deconvoluted the data to extract the pure component spectra while allowing for more accurate identification. The major peaks in each chromatogram were identified *via* a detailed search of the National Institute of Standards and Technology (NIST) mass spectroscopy database, along with information obtained from both the mass spectroscopy fragmentation patterns and the retention indices. The linear retention indices of the compounds were determined by referring to a homologous series of *n*-alkanes (C7–C30).

### 2.5 Semi-quantitative analysis

The relative amounts of each compound were calculated as the percentage of the total peak of all combinations, and these data were analysed with principal components analysis (PCA) using the SPSS 23.0.^14,25^

## 3 Results and discussion

### 3.1 Optimisation of extraction temperature in headspace SPME

The extraction and enrichment of samples by SPME is a dynamic equilibrium process, and the extraction efficiency is related to the distribution coefficient of the analytes between different phases. The distribution coefficient is a thermodynamic constant, and temperature is an important parameter that directly affects the distribution coefficient. Increasing the temperature promotes volatile compounds to reach the headspace and the surface of the extraction fiber. However, the adsorption process between the extraction fiber and the target analyte is generally an exothermic reaction, and high temperature may lead to decreased extraction efficiency and sensitivity. Particularly, excessive temperatures can alter the color of the amber and cause the formation of volatile pyrolysis products from organic polymers. Therefore, when using headspace SPME to extract volatile components from amber, it is necessary to select an appropriate temperature to balance extraction efficiency and the risk of amber pyrolysis. Additionally, the content of volatile components varies with the maturity of the amber. Hence, when extracting amber with a high content of volatile components, the temperature should be appropriately reduced to avoid overextraction, which may result in broadening and overlapping of peaks in two-dimensional chromatography and affect the experimental results. In previous research, the extraction temperature was set at 50 °C, 70 °C, 100 °C (ref. 28) or 80 °C.^14^ In this research, the extraction temperature was divided into five different levels: 40 °C, 60 °C, 80 °C, 100 °C, and 120 °C.

**Fig. 1:**
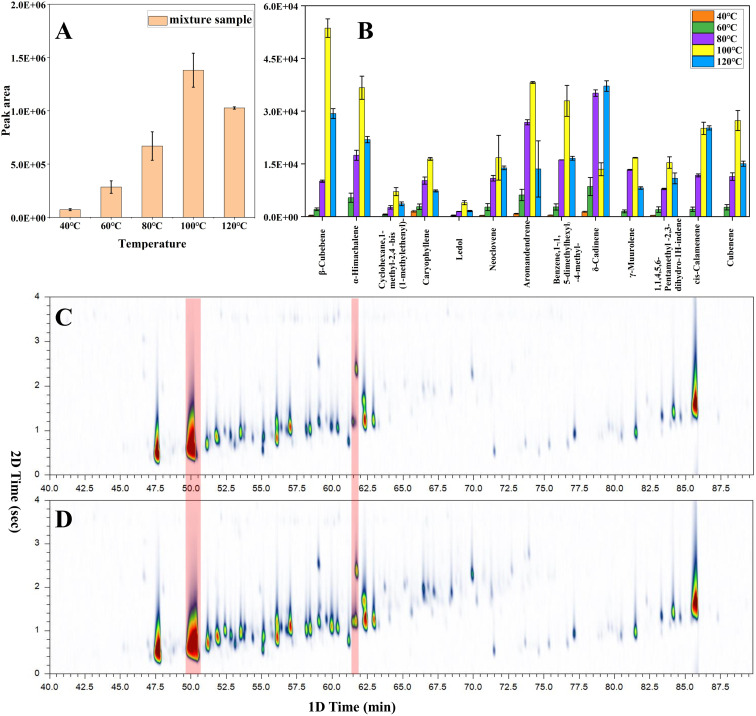
(A) Optimisation of extraction temperature; (B) the comparison of the extraction efficiencies of primary compounds between different temperatures; two-dimensional spectra of mixed samples at (C) 80 °C and (D) 100 °C.

Fig. 1A demonstrates that the maximum extraction efficiency was achieved when the temperature was ramped up to 100 °C. Moreover, the extraction efficiency was reduced when the temperature was increased to 120 °C. Fig. 1B shows that 13 compounds were discovered between the acquired spectrum and the NIST library. These compounds were a little different in content. Some ingredients (α-cubebene, copaene) were removed from the list because their content was much higher than other ingredients. Next, the relative standard deviations (RSD) of the integrated peak area of thirteen compounds were calculated. The RSD (0–19.1%) of the peak area of the main compositions indicate the stability of the samples.

Fig. 1B shows that most compounds display the same trend: the extraction volume increased with an increase in temperature and reached its maximum at 100 °C. Then, this volume decreased with increasing temperature. This is because with the increase of temperature, the distribution coefficient between coating and sample decreases and the equilibrium extraction volume decreases.^32^ When the temperature rises to 100 °C, there are obvious spectral peak over-width and spectral peak superposition phenomena in the two-dimensional chromatography (Fig. 1D), these activities may be attributed to excessive extraction. In order to obtain more accurate experimental results, the extraction temperature of 80 °C (Fig. 1C) is required in this study.

### 3.2 Composition analysis of different producing areas

**Fig. 2:**
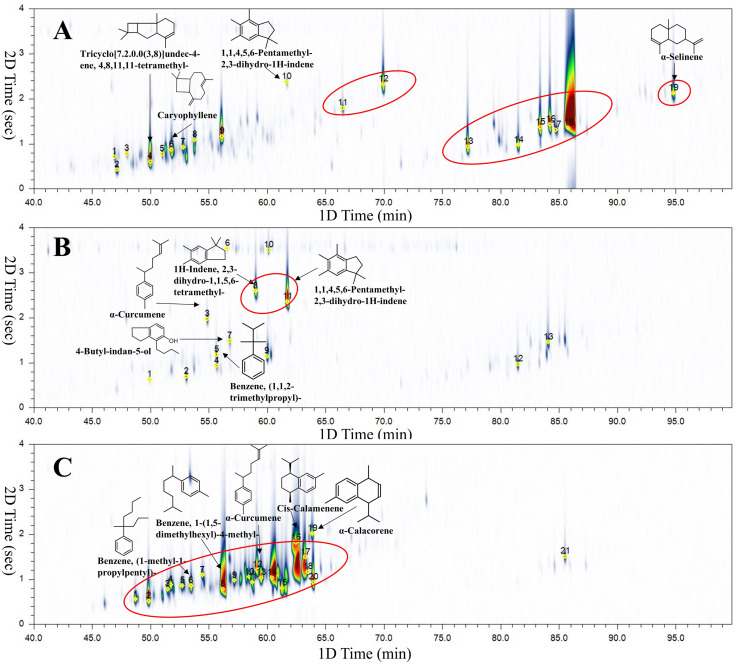
(A) 2D chromatogram of the D1 sample. Peak annotation, see D1 in Table 2; (B) 2D chromatogram of the M10 sample. Peak annotation, see M10 in Table 2; (C) 2D chromatogram of the C2 sample. Peak annotation, see C2 in Table 2.

The samples presented in Table 1 were analysed by HS-SPME-GC × GC-TOFMS. Fig. 2(A–C) depict the chromatograms of representative samples of Dominican amber (D1), Mexican amber (M10), and Colombian copal (C2), respectively. Table 2 summarise these results, and many compounds demonstrated a good match between the acquired spectrum and the NIST library; however, some mass spectra of the compounds could not be accurately assigned.

**Table 2:** HS-SPME-GC × GC-TOFMS results of D1, M10 and C2 samples^a^

No	Compound	1D (min)	2D (s)	CAS	Molecular formula	Match	R.Match	RI	NISTRI	Characteristic mass spectral ions	Sample
1	Ylangene	46.9	0.72	14 912-44-8	C_15_H_24_	844	845	1333	1370	**41(100)**, 105(99), 91(86), 119(72),79(58), 120(40)	**D1**
2	1,6,9-Tetradecatriene	47.1	0.42	61 233-71-4	C_14_H_24_	818	828	1335	—	**41(100)**, 81(65), 67(48), 135(47),55(42), 95(37)	
3	Tricyclo[4.1.0.0(2,4)]heptane,3,3,7,7-tetramethyl-5-(2-methyl-1-propenyl)-	47.9	0.78	56 348-21-1	C_15_H_24_	838	839	1345	—	**41(100)**, 91(86), 161(83), 105(67), 55(40), 133(31)	
4	Tricyclo[7.2.0.0(3,8)]undec-4-ene, 4,8,11,11-tetramethyl-	49.93	0.62	—	C_15_H_24_	917	919	1369	—	**107(100)**, 41(60), 91(50), 133(37),148(36), 80(34)	
5	α-Gurjunene	51	0.769	489-40-7	C_15_H_24_	863	886	1381	1409	**91(100)**, 41(94), 105(88), 119(73), 148(53), 120(41)	
6	Caryophyllene	51.8	0.84	87-44-5	C_15_H_24_	870	882	1391	1419	**41(100)**, 93(77), 79(65), 106(48),67(44), 55(39)	
7	(*Z*,*E*)-α-Farnesene	52.8	0.93	26 560-14-5	C_15_H_24_	853	865	1403	1483	**41(100)**, 119(97), 91(68), 105(52),77(51), 55(36)	
8	α-Himachalene	53.8	1.09	3853-83-6	C_15_H_24_	876	890	1415	1449	**41(100)**, 91(69), 105(62), 77(48), 119(33)	
9	Neoclovene	56.8	1.16	4545-68-0	C_15_H_24_	877	877	1444	1454	**107(100)**, 41(95), 91(73), 122(73),161(70)	
10	1,1,4,5,6-Pentamethyl-2,3-dihydro-1*H*-indene	61.7	2.37	16 204-67-4	C_14_H_20_	883	905	1513	1523	**131(100)**, 173(81), 41(56), 91(44),77(42), 115(36)	
11	Caryophyllene oxide	66.5	1.79	1139-30-6	C_15_H_24_O	823	841	1576	1581	**43(100)**, 55(40), 67(36), 81(28),95(22)	
12	Isolongifolan-8-ol	69.9	2.32	1139-08-8	C_15_H_26_O	817	823	1622	—	**41(100)**, 207(63), 55(59), 123(43),81(37), 95(33)	
13	Tricyclo[4.3.0.0(7,9)]nonane, 2,2,5,5,8,8-hexamethyl-, (1α,6β,7α,9α)-	77.2	0.93	54 832-82-5	C_15_H_26_	764	769	1723	—	**41(100)**, 191(67), 95(58), 69(55),135(42), 107(35)	
14	Cycloheptane, 4-methylene-1-methyl-2-(2-methyl-1-propen-1-yl)-1-vinyl-	81.5	0.97	—	C_15_H_24_	806	823	1786	—	**41(100)**, 55(77),107(62), 93(59),81(52)	
15	Patchoulane	83.4	1.36	25 491-20-7	C_15_H_26_	771	773	1813	—	**41(100)**, 69(23), 95(49), 121(38),55(38), 107(37)	
16	(7*a*-Isopropenyl-4,5-dimethyloctahydroinden-4-yl)methanol	84.3	1.43	—	C_15_H_26_O	756	761	1826	—	**41(100)**, 191(86), 55(51), 95(50), 135(34), 105(30)	
17	Aromadendrene oxide-(1)	84.7	1.31	—	C_15_H_24_O	713	723	1833	—	**41(100)**, 69(47), 95(41), 55(36), 121(35), 107(34)	
18	2,4*a*,5,8*a*-Tetramethyl-1,2,3,4,4*a*,7,8,8*a*-octahydronaphthalen-1-ol	85.8	1.36	20 558-22-9	C_14_H_24_O	675	675	1850	—	**95(100)**, 41(44), 107(34), 55(26),69(18), 121(14)	
19	α-Springene	94.8	2.12	473-13-2	C_15_H_24_	732	769	1990	—	**95(100)**, 41(84), 55(54), 107(49),79(38), 67(34)	
1	Tricyclo[7.2.0.0(3,8)]undec-4-ene, 4,8,11,11-tetramethyl-	49.9	0.6	—	C_15_H_24_	917	919	1369	—	**107(100)**, 41(60), 91(50), 133(37),148(36), 80(34)	**M10**
2	Ledane	53.1	0.70	28 580-43-0	C_15_H_26_	800	801	1406	1373	**41(100)**, 81(83), 67(54), 55(51),107(28)	
3	α-Curcumene	54.9	1.97	644-30-4	C_15_H_22_	728	740	1428	1473	**119(100)**, 132(44), 91(34),41(12)	
4	Longipinane, (*E*)-	55.7	0.93	—	C_15_H_26_	847	850	1438	—	**41(100)**, 109(88), 82(73), 67(58), 55(47)	
5	Benzene,(1,1,2-trimethylpropyl)-	55.7	1.18	26 356-11-6	C_12_H_18_	773	794	1438	—	**119(100)**, 91(32), 41(25), 105(9), 43(1)	
6	Tetradecane	56.6	3.53	629-59-4	C_19_H_40_	872	882	1450	1400	**43(100)**, 57(91), 71(41), 85(28)	
7	4-Butyl-indan-5-ol	56.8	1.48	—	C_13_H_18_O	802	802	1452	—	**147(100)**, 133(16), 115(15)	
8	1*H*-Indene,2,3-dihydro-1,1,5,6-tetramethyl-	59.0	2.59	942-43-8	C_13_H_18_	899	902	1480	—	**159(100)**, 128(27), 115(25), 131(21), 144(17), 77(16)	
9	Caparratriene	60	1.15	—	C_15_H_26_	802	819	1492	1493	**41(100)**, 191(67), 95(58), 69(55), 135(42), 107(35)	
10	Pentadecane	60.1	3.5	629-62-9	C_15_H_32_	843	844	1494	1500	**43(100)**, 57(76), 71(57), 85(30)	
11	1,1,4,5,6-Pentamethyl-2,3-dihydro-1*H*-indene	61.7	2.37	16 204-67-4	C_14_H_20_	883	905	1513	1523	**131(100)**, 173(81), 41(56), 91(44), 77(42), 115(36)	
12	Cycloheptane,4-methylene-1-methyl-2-(2-methyl-1-propen-1-yl)-1-vinyl-	81.5	0.97	—	C_15_H_24_	806	823	1786	—	**41(100)**, 55(77),107(62), 93(59), 81(52)	
13	Dehydrosaussurea lactone	84.1	1.46	28 290-35-9	C_15_H_20_O_2_	760	785	1823	1838	**95(100)**, 41(84), 55(54), 107(49), 79(38), 67(34)	
1	Cyclosativene	48.7	0.56	22 469-52-9	C_15_H_24_	908	915	1354	1368	**105(100)**, 91(92), 119(58), 77(54), 161(39)	**C2**
2	Copaene	4938.0	0.24	3856-25-5	C_15_H_24_	929	932	1367	1376	**105(100)**, 119(76), 91(74), 77(45), 161(37)	
3	Aromandendrene	51.5	0.78	489-39-4	C_15_H_24_	848	870	1387	1440	**41(100)**, 105(99), 91(86), 119(72), 79(58), 120(40)	
4	α-Gurjunene	51.7	0.88	489-40-7	C_15_H_24_	905	921	1390	1409	**91(100)**, 41(94), 105(88), 119(73), 148(53), 120(41)	
5	Isosativene	52.7	0.86	24 959-83-9	C_15_H_24_	915	932	1402	1429	**94(100)**, 41(48), 105(40), 79(33), 119(21), 55(18)	
6	Benzene, (1-methyl-1-propylpentyl)	53.5	0.85	54 932-91-1	C_15_H_24_	754	761	1411	—	**105(100)**, 161(12),77(6)	
7	γ-Gurjunene	54.6	0.93	22 567-17-5	C_15_H_24_	867	881	1425	1473	**41(100)**, 91(82), 105(82), 77(53), 55(40), 119(37)	
8	Benzene, 1-(1,5-dimethylhexyl)-4-methyl	56.2	0.78	1461-02-5	C_15_H_24_	913	935	1445	1448	**119(100)**, 91(25), 105(21), 41(21), 77(11), 204(10)	
9	δ-Cadinene	57.2	0.97	483-76-1	C_15_H_24_	855	882	1470	1516	**105(100)**, 161(73), 91(72), 41(70), 81(57)	
10	γ-Muurolene	58.5	1.05	30 021-74-0	C_15_H_24_	905	914	1473	1477	**41(100)**, 91(91), 105(88), 79(79), 119(60)161(57)	
11	Germacrene D	58.7	0.93	23 986-74-5	C_15_H_24_	863	906	1476	1481	**91(100)**, 105(95), 161(89), 41(77), 79(54), 119(48)	
12	Benzene, 1-(1,5-dimethyl-4-hexenyl)-4-methyl-	59.1	1.21	644-30-4	C_15_H_22_	935	951	1481	1483	**119(100)**, 132(44), 91(34), 41(12)	
13	*cis*-α-Bisabolene	59.5	1.03	29 837-07-8	C_10_H_16_	759	770	1485	1504	**93(100)**, 91(28), 77(28), 105(18), 161(11)	
14	Zonarene	60.4	1.03	41 929-05-9	C_15_H_24_	872	883	1497	1527	**81(100)**, 105(96), 161(76), 41(71), 119(57)	
15	β-Bisabolene	61.3	0.8	495-61-4	C_15_H_24_	929	929	1508	1509	**41(100)**, 69(69), 93(53), 79(32), 91(25), 55(16)	
16	cis-Calamenene	62.5	1.82	483-77-2	C_15_H_22_	862	877	1524	1523	**159(100)**, 128(26), 160(19), 144(14)	
17	Naphthalene, 1,2,3,4,4*a*,7-hexahydro-1,6-dimethyl-4-(1-methylethyl)-	63.3	1.49	16 728-99-7	C_15_H_24_	912	915	1534	1533	**119(100)**, 105(89), 41(45), 161(44), 91(41), 55(24)	
18	α-Muurolene	63.5	1.17	31 983-22-9	C_15_H_24_	898	909	1537	1528	**105(100)**, 41(40), 91(38), 81(30), 161(23), 119(21)	
19	α-Calacorene	63.9	2	21 391-99-1	C_15_H_20_	880	941	1542	1542	**157(100)**, 142(69), 115(26), 128(11), 200(10)	
20	*trans*-α-Bisabolene	63.9	0.93	25 532-79-0	C_15_H_24_	910	934	1543	1512	**93(100)**, 41(50), 79(31), 67(30),	
21	2,4*a*,5,8*a*-Tetramethyl-1,2,3,4,4*a*,7,8,8*a*-octahydronaphthalen-1-ol	85.5	1.51	20 558-22-9	C_14_H_24_O	675	675	1846		**95(100)**, 41(44), 107(34), 55(26), 69(18), 121(14)	

a 1D (min): retention time on first column. 2D(s): retention time on second column. RI: a series of alkanes (C7–C30) was used to calculate the retention indices. NISTRI: relative retention indices taken from NIST17.

An alternative approach was attempted because of the relatively low abundance of these possible marker compounds for discrimination. In total, 29 compounds (see ESI Table S1†) were selected for semi-quantitative analyses. These compounds were sufficiently abundant in most samples and could be unambiguously identified based on the mass spectra and compared with literature data. Peak areas were normalised with respect to the total area of all compounds.

**Fig. 3:**
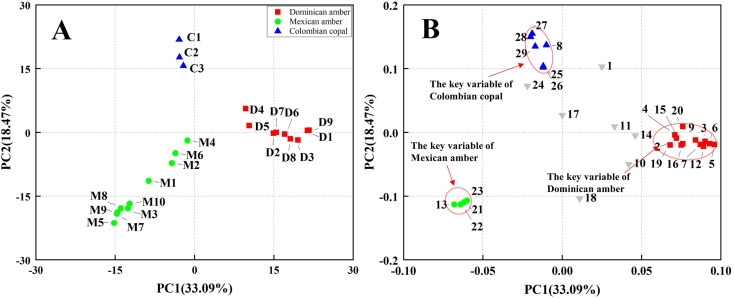
(A) PCA of all samples of amber and copal, classifications based on the relative contents of the different classes of compounds. (B) Variable loadings for components 1 and 2 of the PCA. The four variables identified with green circles define the Mexican amber group, the twelve variables identified with red squares define the Dominican amber group, and the six variables identified with blue triangles define the Colombian copal group.

The compositional data were processed by principal components analysis (PCA). Multivariate statistical techniques of compositional data of ambers have been used to determine the amber types for origin traceability. PCA treatment of the relative abundance data of the 29 selected compounds yielded 29 principal components (PC). The first three PCs accounted for more than 60% of the total variance. The first PC explained 33.09% of the total variance, and the second accounted for 18.47%. These results illustrate that the considered variables were correlated. The PC1–PC2 score plot (Fig. 3A) reveal three distinct groups: Dominican amber, Mexican amber, and Colombian copal. Among these samples, the Colombian copal cluster is present near the origin, and a positive value of PC2 characterises all the samples of Dominican ambers. In contrast, all the Mexican amber samples are located in the positive PC1 area. Fig. 3B reports the projection of the loadings of the different variables on PC1 and PC2. ESI Table S1† lists the components corresponding to each number. The volatile components of the Dominican amber are mainly sesquiterpenoids, for example, Ylangene, β-longipinene, tricyclo [7.2.0.0(3,8)] undec-4-ene,4,8,11,11-tetramethyl-, caryophyllene, α-Springene, ledane, among others. The volatile components of Mexican amber comprise long-chain alkane, sesquiterpenoids, indene compound, such as, 1,1,4,5,6-pentamethyl-2,3-dihydro-1*H*-indene, 1*H*-Indene, 2,3-dihydro-1,1,5,6-tetramethyl-, benzene, (1,1,2-trimethylpropyl), 4-butyl-indan-5-ol. The volatile components of Colombian copal are mainly sesquiterpenoids, such as copaene, α-cubebene, α-muurolene, δ-cadinene, benzene, 1-(1,5-dimethylhexyl)-4-methyl-. Colombian copal has more volatile components than those of Dominican and Mexican amber, which may be attributed to the lower maturity of Columbian copal. Notably, the volatile components of the Dominican amber caryophyllene oxide and isolongifolan-8-ol play an essential role in preventing the invasion of fungi and insects.^33,34^ On the contrary, the volatile component of Colombian Copal, Copaene, mainly acts as an insect attractant,^35^ which may be pollinated by attracting insects, whereas *γ*-gurjunene, a product of biotransformation by plant pathogens,^36^ could also reflect the growth environment of ancient trees to a certain extent.

## 4 Conclusion

Headspace solid-phase microextraction-comprehensive two-dimensional gas chromatography-time-of-flight mass-spectroscopy (HS-SPME-GC × GC-TOFMS) technology was firstly applied to investigate the chemical components (volatile and semi-volatile compositions) and structures of the Dominican amber, Mexican amber, and Colombian copal, which were produced by trees belonging to the genus *Hymenaea* of different places. The critical fingerprints for the origin traceability of amber and copal were analysed *via* principal component analysis for the relative abundances of each compound. Caryophyllene oxide and copaene were only found in Dominican amber and Colombian copal, respectively. 1*H*-Indene,2,3-dihydro-1,1,5,6-tetramethyl- and 1,1,4,5,6-pentamethyl-2,3-dihydro-1*H*-indene were abundantly in Mexican amber, which are the critical fingerprints for the origin traceability of amber and copal produced by trees from the genus *Hymenaea* of different places. The volatile components of these three resinites belong to the low molecular weight volatiles (<250), mainly sesquiterpenoid, oxygenated sesquiterpenoids, indenes, and others, among which the aromatic hydrocarbons are primarily monocyclic or bicyclic aromatic hydrocarbons.

## Conflicts of interest

The authors declare no conflict of interest.

## Supplementary Material

RA-013-D3RA00794D-s001

RA-013-D3RA00794D-s002
