# Supplementary material for: Headspace solid-phase microextraction comprehensive 2D gas chromatography-time of flight mass spectrometry (HS-SPME-GC × GC-TOFMS) for origin traceability of the genus Hymenaea resinites
Source: RSC Adv. 2023 May 9;13(21):14150–8. doi: 10.1039/d3ra00794d (PMC10168135; doi:10.1039/d3ra00794d)

Electronic Supplementary Material (ESI) for RSC Advances.
This journal is © The Royal Society of Chemistry 2023

Supplementary Information

Headspace Solid-phase Microextraction Comprehensive 2D Gas Chromatography–Time of Flight Mass Spectrometry (HS-SPME-GC × GC–TOFMS) for Origin Traceability of the Genus *Hymenaea* Resinites

Xiaopeng SU[#], Jing YU[#], Zhaotong SHI, Yamei WANG[*], Yan LI[*]

[1]*Gemmological Institute, China University of Geosciences, Wuhan 430074, China*

[2]*Hubei Engineering Research Centre of Jewellery, Wuhan 430074, China*

Corresponding authors: yanli@cug.edu.cn; wangym@cug.edu.cn

[#] *These authors contributed equally to the work*

The supplementary data file includes:
Supplementary Table. S1: Key biomarkers for distinguishing the different groups of copal and amber.
Supplementary Fig. S1: 2D Chromatograms from all samples of Dominican amber.
Supplementary Fig. S2: 2D Chromatograms from all samples of Mexican amber.
Supplementary Fig. S3: 2D Chromatograms from all samples of Colombian copal.

Fig. S1: 2D Chromatograms from all samples of Dominican amber.

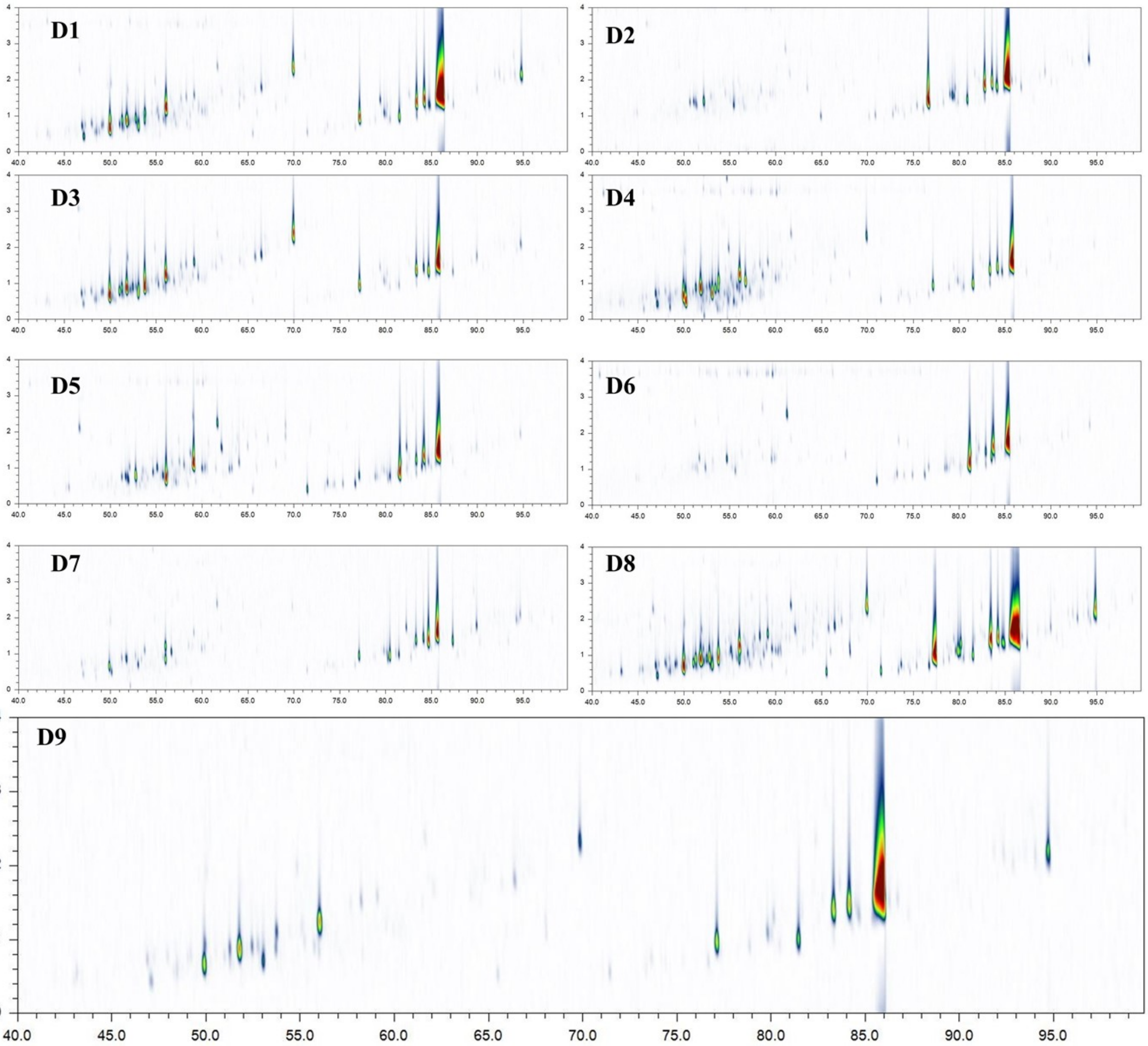

Fig. S2: 2D Chromatograms from all samples of Mexican amber.

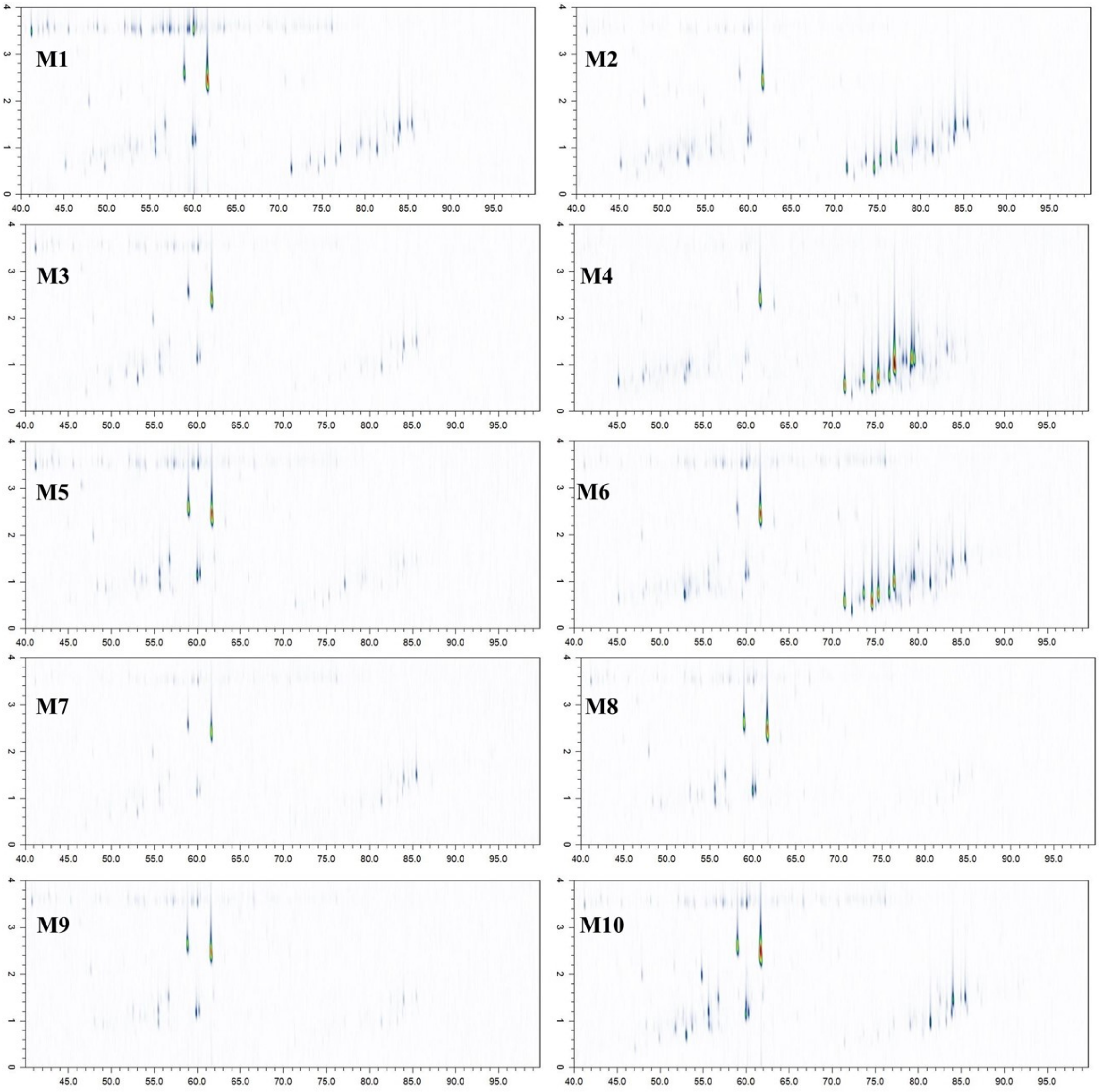

Fig. S3: 2D Chromatograms from all samples of Colombian copal.

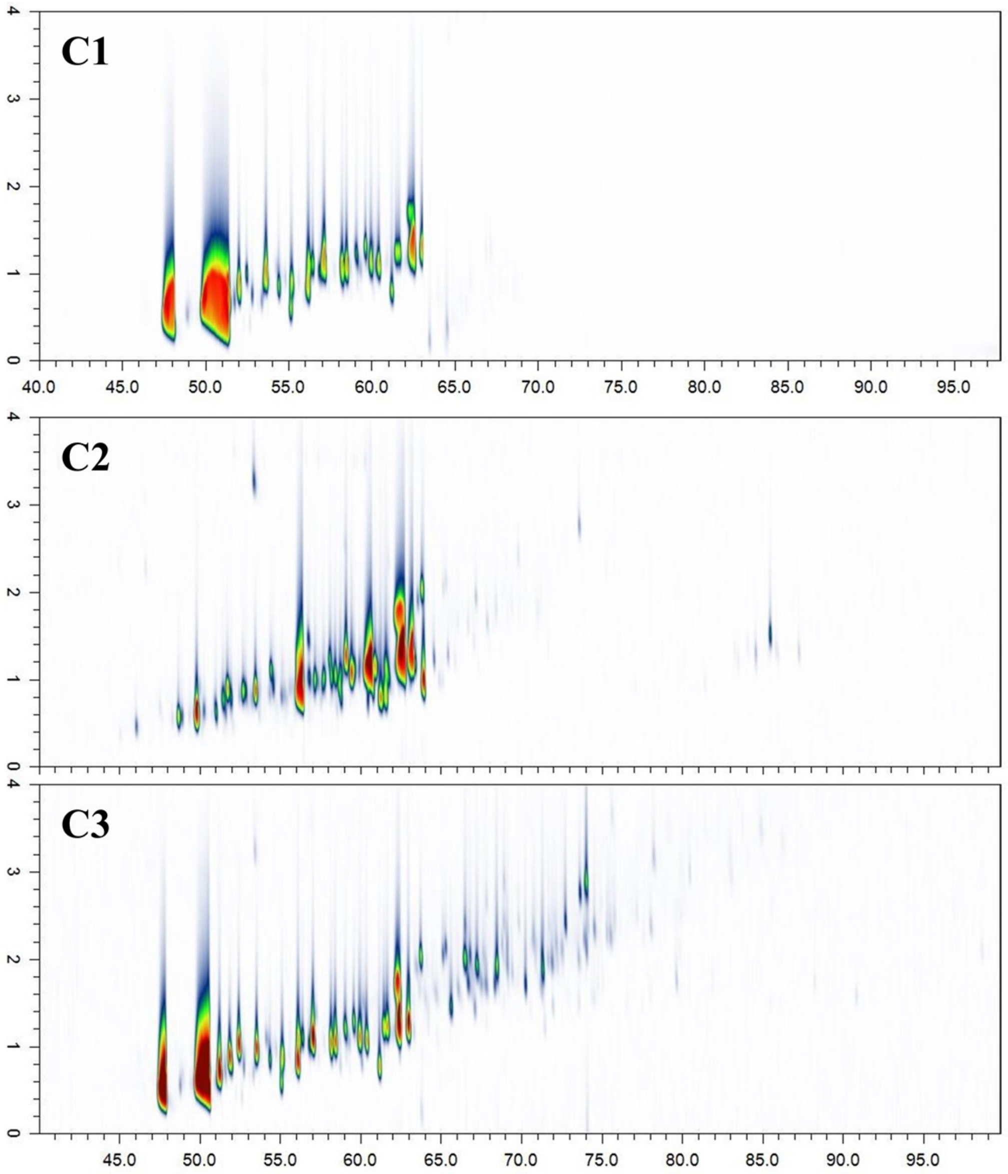

Supplement: RA-013-D3RA00794D-s001 [file RA-013-D3RA00794D-s001.pdf]
